# Use of Generative AI to Identify Helmet Status Among Patients With Micromobility-Related Injuries From Unstructured Clinical Notes

**DOI:** 10.1001/jamanetworkopen.2024.25981

**Published:** 2024-08-13

**Authors:** Kathryn G. Burford, Nicole G. Itzkowitz, Ashley G. Ortega, Julien O. Teitler, Andrew G. Rundle

**Affiliations:** 1 Department of Environmental Health Sciences, Columbia University Mailman School of Public Health, New York; 2 Department of Epidemiology, Columbia University Mailman School of Public Health, New York; 3 Columbia Population Research Center, Columbia University, New York; 4 School of Social Work, Columbia University, New York; 5 Department of Epidemiology, Columbia University Mailman School of Public Health, New York

## Abstract

**Importance:**

Large language models (LLMs) have potential to increase the efficiency of information extraction from unstructured clinical notes in electronic medical records.

**Objective:**

To assess the utility and reliability of an LLM, ChatGPT-4 (OpenAI), to analyze clinical narratives and identify helmet use status of patients injured in micromobility-related accidents.

**Design, Setting, and Participants:**

This cross-sectional study used publicly available, deidentified 2019 to 2022 data from the US Consumer Product Safety Commission’s National Electronic Injury Surveillance System, a nationally representative stratified probability sample of 96 hospitals in the US. Unweighted estimates of e-bike, bicycle, hoverboard, and powered scooter–related injuries that resulted in an emergency department visit were used. Statistical analysis was performed from November 2023 to April 2024.

**Main Outcomes and Measures:**

Patient helmet status (wearing vs not wearing vs unknown) was extracted from clinical narratives using (1) a text string search using researcher-generated text strings and (2) the LLM by prompting the system with low-, intermediate-, and high-detail prompts. The level of agreement between the 2 approaches across all 3 prompts was analyzed using Cohen κ test statistics. Fleiss κ was calculated to measure the test-retest reliability of the high-detail prompt across 5 new chat sessions and days. Performance statistics were calculated by comparing results from the high-detail prompt to classifications of helmet status generated by researchers reading the clinical notes (ie, a criterion standard review).

**Results:**

Among 54 569 clinical notes, moderate (Cohen κ = 0.74 [95% CI, 0.73-0.75) and weak (Cohen κ = 0.53 [95% CI, 0.52-0.54]) agreement were found between the text string–search approach and the LLM for the low- and intermediate-detail prompts, respectively. The high-detail prompt had almost perfect agreement (κ = 1.00 [95% CI, 1.00-1.00]) but required the greatest amount of time to complete. The LLM did not perfectly replicate its analyses across new sessions and days (Fleiss κ = 0.91 across 5 trials; *P* < .001). The LLM often hallucinated and was consistent in replicating its hallucinations. It also showed high validity compared with the criterion standard (n = 400; κ = 0.98 [95% CI, 0.96-1.00]).

**Conclusions and Relevance:**

This study’s findings suggest that although there are efficiency gains for using the LLM to extract information from clinical notes, the inadequate reliability compared with a text string–search approach, hallucinations, and inconsistent performance significantly hinder the potential of the currently available LLM.

## Key Points

**Question:**

Can generative artificial intelligence (AI) be used to identify helmet status of patients injured in micromobility-related accidents from unstructured clinical notes?

**Findings:**

In this cross-sectional study of 54 569 clinical notes, the generative AI large language model (LLM) used in this study had weak to moderate agreement with a text string–search approach for extracting helmet status from deidentified clinical notes unless all researcher-generated text string terms were prompted. The LLM was not able to perfectly replicate its analyses across new sessions and days and did replicate some hallucinations.

**Meaning:**

While there are potential efficiency gains in using the generative AI LLM for information extraction tasks, issues of reliability and hallucinations currently limit its utility.

## Teaser

This cross-sectional study investigates whether an artificial intelligence (AI) large language model can accurately and reliably analyze clinical narratives and identify helmet use status of patients injured in accidents involving e-bikes, bicycles, hoverboards, and powered scooters.

## Introduction

Vast amounts of medically relevant data are included in unstructured clinical narrative notes in electronic medical records (EMR) that are not reflected in the structured data (eg, clinical test data such as blood pressure or *International Statistical Classification of Diseases and Related Health Problems, Tenth Revision [ICD-10]*–coded diagnosis codes) of medical records.^1,2,3^ Information in these clinical notes can be extracted using simple string-matching text search approaches or through more sophisticated artificial intelligence (AI)–based approaches such as natural language processing (NLP).^1,2,3,4,5^ NLP requires that texts be processed in a variety of often time-consuming ways for analysis, including: spelling correction; removal of stop words (eg, “and” and “of”); identification of sentence boundaries; the replacement of inflection forms of words with their base words (Lemmatization); and identification of words with negative modifiers (entity negation).^3,6,7^ The recent development of large language models (LLMs), as exemplified by OpenAI’s ChatGPT (Generative Pre-trained Transformer)^8^ or Meta AI’s LLaMA,^9^ which can simulate a conversation and summarize or classify text, can perhaps present a more efficient, automated way of extracting information from clinical notes in EMRs. LLMs use zero-shot learning, meaning that a prompt-based method is sufficient to extract information rather than relying on labeled training data.^5^

One advantage of string searches and NLP is that they can be accomplished on a desktop computer, with lower risk of inadvertent disclosure of personal health information (PHI); whereas, currently LLMs require that texts be either cut and pasted into a website, such as ChatGPT, or uploaded as a file onto a third-party server. Either manner of submitting clinical notes to a third-party website for analysis by an LLM raises serious research ethic concerns and issues with the Health Information Portability and Accountability Act’s (HIPAA) rules on sharing PHI.^10^ The ethical challenges of using LLMs for health research is likely a primary reason why there are so far very few scientific publications to determine their utility for data mining unstructured clinical narrative notes within large datasets.^11,12,13^ For example, as Zhou and colleagues’ (2023) study data contained PHI, the authors noted they could not use ChatGPT to identify infrequent circumstances in 1462 unstructured reports, but instead implemented an open-source LLM, which did outperform a conventional machine learning approach requiring training data.^13^

As part of our ongoing research into injuries associated with micromobility (ie, bikes, e-bikes, scooters, hoverboard) devices, we use publicly available, deidentified emergency department (ED) clinical notes to explore how LLMs might be applied to unstructured clinical narratives to extract information. We seek to determine whether an LLM can be used to identify the helmet status of patients injured in a micromobility-involved accident at the time of their injury. We compare an LLM to simple text string searches for determining helmet status and measure the test-retest reliability of the LLM. As a secondary objective, we examine the validity of an LLM for determining helmet status among a small random sample.

## Methods

### Data Source

We obtained 2019 through 2022 data from the US Consumer Product Safety Commission’s National Electronic Injury Surveillance System (NEISS) query system, a nationally representative stratified probability sample of 96 hospitals in the US and its territories that contain at least 6 beds and an ED.^14^ NEISS data are publicly available, deidentified, and do not require institutional review board approval or informed consent, in accordance with HIPAA Privacy Rule (45 CFR part 160 and part 164 subparts A and E). This study followed the Strengthening the Reporting of Observational Studies in Epidemiology (STROBE) reporting guideline.

All micromobility-related visits were determined from NEISS product codes and using a text string–search approach for patient narratives used in our ongoing micromobility injury research (eTable 1 in Supplement 1). We included all ED visits among patients with a micromobility-related injury from 2019 to 2022 (n = 54 729 unweighted observations). When there was more than 1 micromobility mode related to the injury, these unweighted records were excluded (n = 160) so that only micromobility modes reported in the first product code variable were included (N = 54 569 unweighted observations).

### Helmet Status Extraction

The Figure depicts an overview of the 2 approaches we used to extract helmet status from unstructured narratives for each patient: (1) text string search and (2) the LLM: ChatGPT-4 (OpenAI).^8^ Prior to implementing the approaches, we operationalized the helmet status variable as wearing helmet vs not wearing helmet vs unknown/not mentioned.

**Figure.**
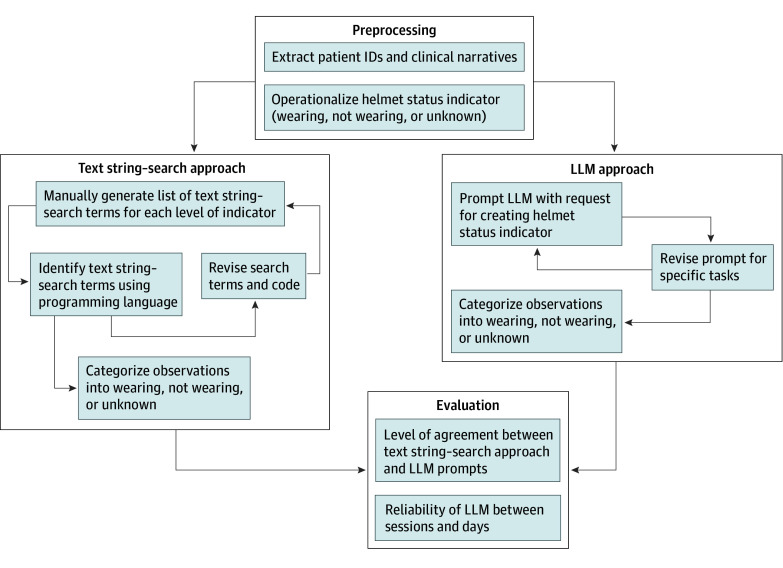
Flowchart for Process and Evaluation Methods for Extracting Information From Patient Clinical Narratives ID indicates identification; LLM, large language model.

#### Text String–Search Approach

We used a text string–search approach applied to patient narratives to categorize helmet status into an indicator variable. We iteratively reviewed the patient narratives to develop a comprehensive list of researcher-generated search terms for each helmet status category (eTable 2 in Supplement 1). Observations with both positive and unknown indicator variables or as both positive and negative indicator variables were coded as unknown helmet use or negative for helmet use, respectively. Observations with both negative and unknown indicator variables were coded as not wearing a helmet. Any observations which did not have an indicator variable for wearing or not wearing a helmet were coded as unknown.

#### LLM Approach

We prompted the LLM^8^ with the datafile containing the patient identification number and associated narrative text, and instructions to generate a helmet status indicator. Multiple chat sessions of prompting the LLM on different days were conducted, with estimates of researcher time to complete each session as shown in Table 1 (for full input and output for each session, see eMethods in Supplement 1). In the first session (November 22, 2023), the prompt merely asked the LLM to classify the narratives as indicating helmet use, no helmet use, and unknown, taking an estimated 5 minutes to complete (low-detail prompt). In the second session (December 7, 2023), all researcher-generated search terms used within the text string–search approach were provided as part of the prompt asking the LLM to classify narratives indicating helmet use, no helmet use, and unknown (high-detail prompt). While little time was required to execute the prompt, it took the researcher an estimated 240 minutes to generate a comprehensive library of search terms. In the last session (December 12, 2023), a prompt with an intermediate level of detail about how to classify the narratives was used (intermediate-detail prompt). To determine the test-retest reliability of the LLM, the high-detail prompt was repeated in 5 new chat sessions on 5 unique days (December 7, 2023; December 8, 2023; December 12, 2023; December 13, 2023; and December 14, 2023).

**Table 1.** Prompts Provided to the LLM to Generate Helmet Status by Date Completed^a^

Date of completion	Estimated time to complete task	Prompt
November 22, 2023 (low-detail prompt)	5 min	“parse through this data looking at column narrative_1 make a table that includes if the cpsc_case_number was wearing a helmet, not wearing a helmet, or if helmet was not mentioned”
December 7, 2023 (high-detail prompt)	240 min	“for every cpsc_case_number there is a narrative_1 with patient description and details of patient accident. for which case_numbers does narrative_1 mention that the case number/patient was wearing/had a helmet at some point? text in narrative_1 to help identify this could include language such as but not limited to: ‘with a helmet’, ‘with helmet’, ‘positive for helmet’, ‘positive for a helmet’, ‘helmeted’, ‘wearing helmet’, ‘wearing a helmet’, ‘w/ helmet’, ‘w helmet’, ‘wore helmet’, ‘w/helmet’, ‘w/ a helmet’, ‘had on a helmet’, ‘had on helmet’, ‘had helmet’, ‘had a helmet’, ‘his helmet’, ‘her helmet’, ‘pt helmet’, ‘pts helmet’, ‘was wearing a helmet’, ‘,helmet,’, ‘helmetd’, ‘whelmet’, ‘cracked helmet’, ‘cracking helmet’, ‘helmet cracked’, ‘helmet on’, ‘broke helmet’, ‘helmet broke’, ‘breaking helmet’, ‘positive helmet’, ‘wore helmet’, ‘wearing a bike helmet’, ‘wearing a full face helmet’, ‘wearing full face helmet’, ‘+helmet’, ‘+ helmet’, ‘including helmet’, ‘ full helmet’, ‘+full helmet’, ‘+ full helmet’, ‘+full bike helmet’, ‘+ full bike helmet’, ‘pt’s helmet’, ‘helmet fell’, ‘smashed helmet’, ‘plus helmet’. ‘helmet was fractured’, ‘helmet was broken’, ‘+ bike helmet’, ‘+bike helmet’, ‘wearing bike helmet’, ‘+ helmemt’, ‘endorses helmet’, ‘helmet went off’, ‘full face mask helmet’, ‘& helmet’, ‘helmet was reported to be cracked’ looking over the initial csv file, for which case_numbers does narrative_1 mention explicitly that the case number/patient was not wearing helmet at any point? text in narrative_1 to help identify this could include language such as but not limited to: ‘no helmet’, ‘without a helmet’, ‘without helmet’, ‘negative for helmet’, ‘negative for a helmet’, ‘not helmeted’, ‘-helmet’, ‘- helmet’, ‘not wearing helmet’, ‘not wearing a helmet’, ‘unhelmeted’, ‘w/o helmet’, ‘did not have on a helmet’, ‘no wearing helmet’, ‘did not have a helmet’, ‘did not have helmet’, ‘w/o a helmet’, ‘without wearing helmet’, ‘without wearing a helmet’, ‘wihtout a helmet’, ‘removed helmet’, ‘denied helmet’, ‘denied use of helmet’, ‘denied use of a helmet’, ‘helmetless’, ‘w/out helmet’, ‘denies helmet’, ‘with out helmet’, ‘with out a helmet’, ‘not wear a helmet’, ‘not wear helmet’, ‘wo a helmet’, ‘not weariing helmet’, ‘not wearinng a helmet’, not wearig’, ‘-helmet’, ‘negative helmet’ looking at the original csv file, for which case_numbers does narrative_1 not mention helmet(s) or mention helmet use unknown? text in narrative_1 to help identify this could include language such as but not limited to: ‘unsure if helmet’, ‘unsure if helmeted’, ‘unsure if pt wearing helmet’, ‘unsure if pt wearing a helmet’, ‘unsure if pt was wearing helmet’, ‘unsure if pt was wearing a helmet’, ‘unknown if helmet’, ‘helmet unknown’, ‘unknown helmet’, ‘no mention of helmet’, ‘unk helmet’, ‘helmet unk’, ‘helmet ns’, ‘ns helmet’, ‘?helmet’, ‘? helmet’, ‘helmet?’ could you make a csv file with cpsc_case_number, narrative_1, and new column “helmet status”. based on their narrative_1 descriptions and criteria above categorize each case number “helmet not mentioned” or “not wearing helmet” or “wearing helmet” for all case_numbers. there should be no duplicate case numbers. should be one-to-one. double check for mistakes”
December 12, 2023 (intermediate-detail prompt)	15 min	“for every cpsc_case_number there is a narrative_1 with patient description and details of a patient accident that involved an injury. based on their narrative_1 descriptions, can you use the following criteria to create a new variable called “helmet_status”, which is generated by categorizing each case number into one of the following categories “helmet not mentioned” or “not wearing helmet” or “wearing helmet” using these criteria: “helmet not mentioned”: cpsc_case_numbers where narrative_1 mentions that helmet use was unknown or that the term “helmet” was not recorded within narrative_1. the narrative_1 column could include any variation of the following phrases: “unsure if helmeted”, “unknown if helmeted”, “no mention of helmet”, or “?helmet”. please consider that there could be other phrases that indicate unknown helmet use. “not wearing helmet”: cpsc_case_numbers where narrative_1 mentions that the case number/patient was not wearing a helmet at any point. the narrative_1 column could include any variation of the following phrases: “no helmet” or “without helmet” or “not wearing helmet” or “unhelmeted”. please consider that there could be other phrases that indicate the patient was not wearing a helmet. “wearing helmet”: cpsc_case_numbers where narrative_1 mentions that the case number/patient was wearing/had a helmet at some point. the narrative_1 column could include any variation of the following phrases: “with a helmet” or “had a helmet” or “cracked helmet” or “+ helmet”. please consider that there could be other phrases that indicate the patient was wearing a helmet. can you make a csv file with cpsc_case_number, narrative_1, and new column “helmet status” based on the criteria above?”

Abbreviation: LLM, large language model.

^a^ See eMethods in Supplement 1 for library of text strings used for string-matching and the LLM prompt.

#### Criterion Standard Approach

To create a criterion standard set of ratings based on human review of the clinical notes, authors (K.B., N.I., and A.R.) reviewed a stratified random sample of 400 clinical notes from the high-detail prompt conducted on December 7, 2023. Each author reviewed and coded a third of unique records plus 100 records assigned to all 3 authors. There was 100% agreement across all 3 reviewers on the jointly coded records. We hypothesized that the unknown category might contain information about helmet use that both the text string searches and the LLM missed, and thus oversampled records classified as unknown. There were 200 records drawn from the unknown category, and 100 each from the wearing helmet and not wearing helmet categories.

### Statistical Analysis

Contingency tables and Cohen κ test statistics with 95% CIs were calculated to assess the level of agreement between the text string–search approach and the 3 LLM prompts for generating helmet status. Fleiss κ was calculated to assess the test-retest reliability of the helmet status variable generated using the high-detail prompt (prompt shown in Table 1B) across all 5 sessions that occurred on 5 separate days. κ statistics were interpreted as: almost perfect (>0.90), strong (0.80-0.90), moderate (0.60-0.79), weak (0.40-0.59), minimal (0.21-0.39), and none (0-0.20).^15^ To evaluate the performance of the 2 approaches with the criterion standard sample, recall, precision, F1 score, and Cohen κ statistics were calculated.^16^ Statistical analyses were performed using R statistical software version 4.3.1 (R Core Team 2023) from November 2023 to April 2024. The significance level was set at 2-tailed *P* < .05. Data and full reproducible code are available to access in Zenodo.

## Results

The LLM and text string–search matching were used to extract and categorize helmet status from 54 569 clinical notes. Table 2 displays the contingency tables and level of agreement for the helmet status variable between 3 LLM sessions and the text string–search approach. There was moderate agreement (κ = 0.74 [95% CI, 0.73-0.75]) between the LLM with no search terms provided within the low-detail prompt (Table 1) and string-search to generate helmet status. Upon examination of the disagreements between the 2 approaches, the LLM misclassified terms that were categorized into the unknown category (n = 995) such as “helmet ns” (n = 454) and “unknown” (n = 84) or regular expression terms such as “?helmet” (n = 125). However, there were also terms in which both approaches misclassified terms, “-loc or helmet use” (n = 1), or that were not identified for use in the text string–search approach, “helmet was cracked” (n = 4). The greatest disagreement between the 2 approaches was for the LLM’s classification of a patient as wearing a helmet compared with the text string–search approach classifying the patient as not wearing a helmet (n = 4072). The mismatch was largely due to the LLM errors in identifying negations such as “without a helmet” (n = 278), “w/o helmet” (n = 543), “unhelmeted” (n = 1594), “not wearing helmet” (n = 197), or regular expression terms (“-helmet”; n = 276).

**Table 2.** Agreement Between the LLM and Text String Search for Helmet Status (N = 54 569)^a^

LLM approach^c^	Text string–search approach^b^				Cohen κ (95% CI)
	Wearing helmet	Not wearing helmet	Unknown	Total	
**Low-detail prompt (November 22, 2023)**					
Wearing helmet	5081	4072	995	10 148	0.74 (0.73-0.75)
Not wearing helmet	0	1908	0	1908	
Unknown	1	0	42 512	42 513	
Total	5082	5980	43 507	54 569	
**High-detail prompt (December 7, 2023)**					
Wearing helmet	5081	0	29	5110	1.00 (1.00-1.00)
Not wearing helmet	0	5980	1	5981	
Unknown	1	0	43 477	43 478	
Total	5082	5980	43 507	54 569	
**Intermediate-detail prompt (December 12, 2023)**					
Wearing helmet	532	0	0	532	0.53 (0.52-0.54)
Not wearing helmet	0	3856	0	3856	
Unknown	4550	2124	43 507	50 181	
Total	5082	5980	43 507	54 569	

Abbreviation: LLM, large language model.

^a^ Counts (No.) provided within cells.

^b^ See eTable 1 in Supplement 1 for library of text strings used for string-matching and LLM prompt.

^c^ See Table 1 for prompt provided to the LLM.

Table 2 shows a nearly perfect agreement between the LLM using the high-detail prompt, with all researcher-generated search terms provided in the prompt, and the text string–search approach using identical search terms (κ = 1.00 [95% CI, 1.00-1.00]). For the 29 instances where the LLM identified the patient wearing a helmet, and the text string–search approach determined helmet use was unknown, the LLM did not extract the following terms: “unknown” (n = 3), “unsure if pt was wearing a helmet” (n = 8), “no mention of helmet” (n = 2), “helmet unk” (n = 1), and regular expression term “?helmet” (n = 15). However, the text string–search approach misclassified 1 record as unknown, as 1 spelling error term, “was not wearig a helmet”, was not included in the library of researcher-defined terms. The LLM also did not identify one spelling error with a regular expression term “+helmemt” resulting in the misclassification as unknown.

There was weak agreement (Cohen κ = 0.53 [95% CI, 0.52-0.54]) between the string search approach and the LLM using the intermediate-detail prompt (Table 1). For the 4550 cases in which there was disagreement between the text string–search approach and the LLM for helmet status, the LLM did not extract terms indicating the patient was wearing a helmet including “helmeted” (n = 1614), “wearing a helmet” (n = 865), “wearing helmet” (n = 575), “with helmet” (n = 161), “w/helmet” (n = 40). The LLM also failed to extract negated terms, such as “w/o helmet” (n = 542), “without helmet”; (n = 278) indicating a patient was not wearing a helmet, rather characterizing these instances as unknown, which resulted in 2124 disagreements.

Table 3 shows the results of the test-retest reliability for the LLM using the high-detail prompt across 5 unique sessions and days (Table 1). The test-retest reliability was high (Fleiss κ = 0.91; *P* < .001), although the κ score was largely driven by agreement on the “unknown” category. Results from sessions 2, 3, and 4 had perfect agreement with each other on helmet status categorization. However, the results from these 3 sessions had 2902 and 2900 instances of disagreements with session 1 and 5, respectively. After inspection of these disagreements, sessions 2 to 4 misclassified negated helmet terms, such as “w/o helmet”, “unhelmeted”, and “not wearing helmet” as the patient wearing a helmet. Regarding the difference in results for sessions 1 and 5, in session 1 a spelling error for regular expression (“+helmemt”) was misclassified as unknown, which was identified in session 5 correctly as wearing a helmet. However, in session 5, a spelling error (“not wearig a helmet”) was incorrectly classified as unknown but correctly classified in session 1 as not wearing a helmet.

**Table 3.** Test-Retest Reliability for the LLM for High-Detail Prompt Across 5 Sessions and Days^a^^,^^b^

	Session 1 (December 7, 2023)	Session 2 (December 8, 2023)	Session 3 (December 12, 2023)	Session 4 (December 13, 2023)	Session 5 (December 14, 2023)
Helmet status					
Wearing helmet	5110	8011	8011	8011	5111
Not wearing helmet	5981	3080	3080	3080	5980
Unknown	43 478	43 478	43 478	43 478	43 478
Fleiss κ (*P* value)	0.91 (<.001)^c^				

Abbreviation: LLM, large language model.

^a^ Counts (No.) provided within cells.

^b^ See Table 1 for prompt provided to the LLM.

^c^ This Fleiss κ (*P* value) was calculated across all 5 sessions.

Table 4 reports on the performance of the LLM and the text string search compared with the human-coded criterion standard results for a random subsample of records. Results were identical across approaches, with very high validity (κ = 0.98 [95% CI, 0.96-1.00]), and very high precision, recall, and F1 scores (all between 0.962 to 1.0) for the classification of helmet status. In total, there were 6 misclassifications by the LLM and the text string search, which included 4 records for not wearing a helmet being incorrectly classified as either unknown or wearing a helmet, and 2 records for wearing a helmet being incorrectly classified as unknown.

**Table 4.** Performance of the LLM and Text String Search Compared With Criterion Standard (n = 400)^a^

	Precision			Recall			F1 Score			Cohen κ (95% CI)
	Wearing helmet	Not wearing helmet	Unknown	Wearing helmet	Not wearing helmet	Recall unknown	Wearing helmet	Not wearing helmet	Unknown	
LLM	0.98	1.00	0.98	0.98	0.96	1.00	0.98	0.980	1.00	0.98 (0.96-1.00)
Text string search	0.98	1.00	0.98	0.98	0.96	1.00	0.98	0.98	1.00	0.98 (0.96-1.00)

Abbreviation: LLM, large language model.

^a^ These 400 records were a random draw from the high-detail prompt (December 7, 2023).

## Discussion

This study examined the potential of an LLM for extracting information from clinical notes, a rich source of information for health professionals and researchers, within a large publicly available dataset. For prompts that did not include the search terms used within the text string–search approach (low-detail prompt), the LLM had moderate agreement with the text string–search approach. The discrepancies were largely due to the inability of the LLM used in this study to identify negations (“unhelmeted”, “w/o helmet”) and unknown instances (“helmet ns”). When some of the text string–search terms were prompted to the LLM (intermediate-detail prompt), the result was a lower agreement with the text string–search approach than produced by the low-detail prompt. These disagreements were primarily due to the LLM’s misclassifications of both wearing and not wearing a helmet. However, when the exact search terms used in the text string search were provided to the LLM (high-detail prompt), agreement with the text string searches was almost perfect (December 7, 2023). When the results of these LLM sessions were compared with the criterion standard data, both the LLM and the text string search showed very high validity. However, the test-retest reliability analyses of using the high-detail prompt showed that the LLM was not able to replicate its analyses and was more consistent in replicating its hallucinations (ie, not wearing helmet classified as wearing helmet) than accurate coding. These are major methodological concerns for the routine use of the LLM used in this study for analyzing clinical notes.

These findings suggest that the LLM we used did not outperform a basic approach for data extraction of clinical narrative notes from a large dataset. The system only worked well when prompted with all of the text strings used in the text search, essentially negating the envisioned efficiency (eg, time, human labor) gains of using LLMs. This may be explained by the high level of detail in this prompt preventing the LLM from having to guess as it did in the low- and intermediate-detail prompts.^17^ However, it was interesting that the intermediate-detail prompt, even with more instruction, performed worse than the low-detail prompt. As the LLM used in this study is also found to perform better on simple tasks,^17^ the combination of a complex prompt that also causes the LLM to guess on the task could explain the poorest performance on the intermediate-detail prompt. Alternatively, given the inconsistency of the LLM across sessions, the low performance with the intermediate-detail prompt might just be an instance of the LLM we used in this study having an off day.

A major limitation for using the LLM in the information extraction task was the misclassification of negated and unknown/not specified phrases unless prompted, which largely contributed to the poor reliability with the basic string-search method. This was further demonstrated in the ground truth analysis, which resulted in 6 misclassifications due to terms not being found for inclusion in the original library of prompted terms. A further concern of the LLM used in this study is the inconsistency in results across new sessions and days, even when prompted with the exact search terms to extract.

Evidence shows that LLMs perform worse on negated instruction prompts compared with prompts that are not negated, and relative to humans on tasks containing negations.^18^ Additionally, the LLM used in this study has demonstrated several unreliable behaviors including the ability to generate the same predictions on similar text inputs.^19,20^ While one strategy to address inconsistent responses on tasks is to determine the optimal prompt through human finetuning, researchers question this possibility as LLMs may be pretrained on information deriving from different sources.^19,21^ There are emerging best practices for prompt engineering that could be leveraged to improve performance on tasks including iterative testing and human finetuning,^17^ or more advanced strategies such as temperature and token control to generate more focused or creative responses.^22^ The most optimal approach for extracting reliable information is through finetuning LLMs for specific tasks using labeled data sources but this would undermine the efficiencies of using this approach.^23^

While the LLM did not perform as well as a basic method for extracting and categorizing helmet status in the present study, it was able to identify a spelling error (“was not wearig a helmet”) and search terms that were not included in the researcher-generated library of text strings (eg, “helmet was cracked”). The text string–search approach required a time-consuming process of a human researcher iteratively reviewing all possible text strings, in which mistakes may be inevitable. Furthermore, the LLM used in this study does not require advanced data science skills as required by NLP techniques, which may lead to reduced costs and time for research and clinical teams.^24,25^

### Limitations

There are limitations to this study. First, we did not determine the ground truth for helmet status for all 54 569 clinical narratives through human review. This resulted in several misclassifications by the text string–search approach, which was further indicated in the ground truth analysis. It is possible that there were other instances, particularly when both approaches reported helmet status as unknown, where the results of text string searches and the LLM agreed with each other, but both approaches produced the incorrect answer. Given the size of the dataset, human error in seeking the ground truth of the full dataset would be inevitable. Second, the findings cannot be generalized to all datasets, which may vary in their degrees of complexity (eg, spelling errors, negation terms). Additionally, the prompts used within the present study may not be optimal for the desired task (Table 1). OpenAI released a prompt engineering guide after analyses were performed in this study with strategies for improving results from the LLM we used.^17^ Future work should systematically evaluate those strategies for specific tasks and with reference to criterion standards, as performed within this study, to establish best practices.

## Conclusions

For information extraction from clinical notes, the LLM used in this study had moderate to weak agreement with a simple string search method, unless all of the human-labeled text strings were provided as part of the prompt. As each existing information extraction approach has major limitations,^5,26,27^ combining LLMs, rule-based system approaches, and NLP techniques will perhaps result in the most effective method for data extraction from clinical notes. We encourage others to use these deidentified, publicly available data to gleam other beneficial use cases of this rapidly developing AI system for health professionals.

## Notes

Supplement 1. **eTable 1.** Product Codes and Text-String Terms Used to Generate Micromobility Variables in the National Electronic Injury Surveillance System Data, 2019-2022 **eTable 2.** Researcher-Generated Text-String Terms for “Helmet” Indicator Within 2019-2022 NEISS Dataset **eMethods.** Comprehensive Inputs (Prompts) and Outputs From ChatGPT-4 Sessions

Supplement 2.
Data Sharing Statement
