# Supplementary material for: Use of Generative AI to Identify Helmet Status Among Patients With Micromobility-Related Injuries From Unstructured Clinical Notes
Source: JAMA Netw Open. 2024 Aug 13;7(8):e2425981. doi: 10.1001/jamanetworkopen.2024.25981 (PMC11322845; doi:10.1001/jamanetworkopen.2024.25981)
Supplement: Supplement 1. — eTable 1. Product Codes and Text-String Terms Used to Generate Micromobility Variables in the National Electronic Injury Surveillance System Data, 2019-2022 eTable 2. Researcher-Generated Text-String Terms for “Helmet” Indicator Within 2019-2022 NEISS Dataset eMethods. Comprehensive Inputs (Prompts) and Outputs From ChatGPT-4 Sessions [file jamanetwopen-e2425981-s001.pdf]

Supplemental Online Content

Burford KG, Itzkowitz NG, Ortega AG, Teitler JO, Rundle AG. Use of generative AI to identify helmet status among patients with micromobility-related injuries from unstructured clinical notes. *JAMA Netw Open.* 2024;7(8):e2425981. doi:10.1001/jamanetworkopen.2024.25981

eTable 1. Product Codes and Text-String Terms Used to Generate Micromobility Variables in the National Electronic Injury Surveillance System Data, 2019-2022

eTable 2. Researcher-Generated Text-String Terms for "Helmet" Indicator Within 2019-2022 NEISS Dataset

eMethods. Comprehensive Inputs (Prompts) and Outputs From ChatGPT-4 Sessions

This supplemental material has been provided by the authors to give readers additional information about their work.

© 2024 Burford KG et al. *JAMA Network Open.*

eTable 1. Product codes and text-string terms used to generate micromobility variables in the National Electronic Injury Surveillance System data, 2019-2022.[a]

Micromobility mode	Product codes, 2019	Product codes, 2020-2022	Narrative text search terms
Powered scooter	5042 (scooters/skateboards, powered)	5022 (rideshare or rental scooter or scooters, powered)	‘scooter’
Hoverboard	5042 (standup scooter/skateboard, powered)	5025 (hoverboards and powered skateboards)	‘hoverboard’ or ‘hooverboard’ or ‘hover board’ or ‘hoover board’ or ‘hoverbaord—hooverbaord’ or ‘hover boad’ or ‘hoverboad’ or hover
E-bike	5035 (minibikes, powered)<br>3215 (mopeds or power-assisted cycles)	5035 (minibikes, powered)<br>3215 (electric bikes (e-bikes) or mopeds or power-assisted cycles)	'elect' or 'batt' or 'motor' and 'bike' or 'bicycle'
Bicycle	5040 (bicycles or accessories (excl. mountain or all-terrain bicycles)<br>5033 (mountain or all-terrain bicycles or accessories)	5040 (bicycles or accessories (excl. mountain or all-terrain bicycles)<br>5033 (mountain or all-terrain bicycles or accessories)	

© 2024 Burford KG et al. *JAMA Network Open.*

eTable 2. Researcher-generated text-string terms for “helmet” indicator within 2019-2022 NEISS dataset.[a]

Helmet Indicator	Search Terms
Not wearing helmet	"NO HELMET", "WITHOUT A HELMET", "WITHOUT HELMET", "NEGATIVE FOR HELMET", "NEGATIVE FOR A HELMET", "NOT HELMETED", "-HELMET", "- HELMET", "NOT WEARING HELMET", "NOT WEARING A HELMET", "UNHELMETED", "W/O HELMET", "DID NOT HAVE ON A HELMET", "NO WEARING HELMET", "DID NOT HAVE A HELMET", "DID NOT HAVE HELMET", "W/O A HELMET","WITHOUT WEARING HELMET", "WITHOUT WEARING A HELMET", "REMOVED HELMET", "DENIED HELMET", "DENIED USE OF HELMET", "DENIED USE OF A HELMET", "HELMETLESS", "W/OUT HELMET", "DENIES HELMET", "WITH OUT HELMET", "WITH OUT A HELMET", "NOT WEAR A HELMET", "NOT WEAR HELMET", "WO A HELMET", "-HELMET", "NEGATIVE HELMET", "WIHTOUT A HELMET", "NOT WEARIING HELMET", "NOT WEARINNG A HELMET", "NOT WEARIG HELMET"
Wearing helmet	"WITH A HELMET", "WITH HELMET", "POSITIVE FOR HELMET", "POSITIVE FOR A HELMET", "HELMETED", "WEARING HELMET", "WEARING A HELMET", "W/ HELMET", "W HELMET", "WORE HELMET", "W/HELMET", "W/ A HELMET", "HAD ON A HELMET", "HAD ON HELMET", "HAD HELMET", "HAD A HELMET", "HIS HELMET", "HER HELMET", "PT HELMET", "PTS HELMET", "WAS WEARING A HELMET", ",HELMET,", "CRACKED HELMET", "CRACKING HELMET", "HELMET CRACKED", "HELMET ON", "BROKE HELMET", "HELMET BROKE", "BREAKING HELMET", "POSITIVE HELMET", "WORE HELMET", "WEARING A BIKE HELMET", "WEARING A FULL FACE HELMET", "WEARING FULL FACE HELMET", "INCLUDING HELMET", " FULL HELMET", "PT'S HELMET", "HELMET FELL", "SMASHED HELMET", "PLUS HELMET", "HELMET WAS FRACTURED", "HELMET WAS BROKEN", "WEARING BIKE HELMET", "ENDORSES HELMET", "HELMET WENT OFF", "FULL FACE MASK HELMET", "& HELMET", "HELMET WAS REPORTED TO BE CRACKED", "HELMETD", "WHELMET", “+HELMET”, “+ HELMET”, “+FULL HELMET”, “+ FULL HELMET”, “+FULL BIKE HELMET”, “+ FULL BIKE HELMET”, “+BIKE HELMET”, “+ BIKE HELMET”, “& HELMET”, “&HELMET”, “+HELMEMT”, “+ HELMEMT”
Unknown	"UNSURE IF HELMET", "UNSURE IF HELMETED", "UNSURE IF PT WEARING HELMET", "UNSURE IF PT WEARING A HELMET", "UNSURE IF PT WAS WEARING HELMET", "UNSURE IF PT WAS WEARING A HELMET", "UNKNOWN IF HELMET", "HELMET UNKNOWN", "UNKNOWN HELMET", "NO MENTION OF HELMET", "UNK HELMET", "HELMET UNK", "HELMET NS", "NS HELMET", “? HELMET”, “?HELMET”, “HELMET?”

[a]NEISS: National Electronic Injury Surveillance System

© 2024 Burford KG et al. *JAMA Network Open.*

eMethods. Comprehensive inputs (prompts) and outputs from ChatGPT-4 sessions.

November, 22 2023 (Low Detail Prompt)

You
Spreadsheet
Parse through this data looking at column narrative_1 make a table that includes if the cpsc_case_number was wearing a helmet, not wearing a helmet, or if helmet was not mentioned

ChatGPT
The dataset contains two columns: cpsc_case_number and narrative_1. To create a new table that includes information about whether a helmet was worn, not worn, or not mentioned in narrative_1 column, I'll parse through the narratives and categorize each case accordingly. Let's proceed with the analysis.

The new table has been created, which includes a column helmet_use_category categorizing each case as "Wearing helmet, 'Not wearing Helmet', or 'Helmet not mentioned', based on the information provided in the narrative_1 column. The first few rows of the updated dataset show that in these instances, the helmet was not mentioned. If you need any further analysis or a different view of this data, please let me know.

You
Where is the table?

ChatGPT
The updated table with the helmet_use_category has been saved as a CSV file. You can download it using the link below:
Download the Updated Table

© 2024 Burford KG et al. *JAMA Network Open.*

December 07, 2023 (High Detail Prompt)

You
Spreadsheet
Here is a spreadsheet we will be working with.

ChatGPT
The spreadsheet contains data on bicycle-related incidents. Here are the columns in the dataset:

1. **Unnamed: 0**: This appears to be an index or identifier for each row.
2. **cpsc_case_number**: This column likely contains a unique case number for each incident.
3. **narrative_1**: This column contains narratives or descriptions of the incidents.

Each row seems to represent a different incident involving bicycles, with details like the age and gender of the person involved, the nature of the incident, and whether a helmet was used.
How would you like to proceed with this data? Do you have any specific analysis or questions in mind?

You
For every cpsc_case_number there is a narrative_1 with patient description and details of patient accident. For which case_numbers does Narrative_1 mention that the case number/patient was wearing/had a helmet at some point? Text in Narrative_1 to help identify this could include language such as but not limited to: 'WITH A HELMET', 'WITH HELMET', 'POSITIVE FOR HELMET', 'POSITIVE FOR A HELMET', 'HELMETED', 'WEARING HELMET', 'WEARING A HELMET', 'W/ HELMET', 'W HELMET', 'WORE HELMET', 'W/HELMET', 'W/ A HELMET', 'HAD ON A HELMET', 'HAD ON HELMET', 'HAD HELMET', 'HAD A HELMET', 'HIS HELMET', 'HER HELMET', 'PT HELMET', 'PTS HELMET', 'WAS WEARING A HELMET', ',HELMET,', 'HELMETD', 'WHELMET', 'CRACKED HELMET', 'CRACKING HELMET', 'HELMET CRACKED', 'HELMET ON', 'BROKE HELMET', 'HELMET BROKE', 'BREAKING HELMET', 'POSITIVE HELMET', 'WORE HELMET', 'WEARING A BIKE HELMET', 'WEARING A FULL FACE HELMET', 'WEARING FULL FACE HELMET', '+HELMET', '+ HELMET', 'INCLUDING HELMET', ' FULL HELMET', '+FULL HELMET', '+ FULL HELMET', '+FULL BIKE HELMET', '+ FULL BIKE HELMET', 'PT'S HELMET', 'HELMET FELL', 'SMASHED HELMET', 'PLUS HELMET'. 'HELMET WAS FRACTURED', 'HELMET WAS BROKEN', '+ BIKE HELMET', '+BIKE HELMET', 'WEARING BIKE HELMET', '+ HELMEMT', 'ENDORSES HELMET', 'HELMET WENT OFF', 'FULL FACE MASK HELMET', '& HELMET', 'HELMET WAS REPORTED TO BE CRACKED'
ChatGPT
ChatGPT
Error analyzing
It appears that the regular expression used to search for the specified keywords in the narratives encountered an error due to a special character or sequence. This issue can happen with characters like '+', '*', '?', etc., which have special meanings in regular expressions. I will adjust the search method to handle these special characters correctly and then proceed to identify the case numbers. Let's try again.
I have identified the case numbers where the narrative mentions that the individual was wearing a helmet or had a helmet at some point. Here are the first 10 such case numbers:

4. 190106883
5. 190113371

© 2024 Burford KG et al. *JAMA Network Open.*

6. 190114894
7. 190119860
8. 190130002
9. 190131680
10. 190131715
11. 190136254
12. 190137294
13. 190139436

In total, there are 8,032 cases in your dataset where the narrative includes a mention of a helmet.

You

Looking over the initial csv file, for which case_numbers does Narrative_1 mention explicitly that the case number/patient was not wearing helmet at any point? Text in narrative_1 to help identify this could include language such as but not limited to : 'NO HELMET', 'WITHOUT A HELMET', 'WITHOUT HELMET', 'NEGATIVE FOR HELMET', 'NEGATIVE FOR A HELMET', 'NOT HELMETED', '-HELMET', '- HELMET', 'NOT WEARING HELMET', 'NOT WEARING A HELMET', 'UNHELMETED', 'W/O HELMET', 'DID NOT HAVE ON A HELMET', 'NO WEARING HELMET', 'DID NOT HAVE A HELMET', 'DID NOT HAVE HELMET', 'W/O A HELMET', 'WITHOUT WEARING HELMET', 'WITHOUT WEARING A HELMET', 'WIHTOUT A HELMET', 'REMOVED HELMET', 'DENIED HELMET', 'DENIED USE OF HELMET', 'DENIED USE OF A HELMET', 'HELMETLESS', 'W/OUT HELMET', 'DENIES HELMET', 'WITH OUT HELMET', 'WITH OUT A HELMET', 'NOT WEAR A HELMET', ‘NOT WEAR HELMET’, ‘WO A HELMET’, ‘NOT WEARIING HELMET’, ‘NOT WEARINNG A HELMET’, NOT WEARIG’, ‘-HELMET’, ‘NEGATIVE HELMET’

ChatGPT

I have identified the case numbers where the narrative explicitly mentions that the individual was not wearing a helmet at any point. Here are the first 10 such case numbers:

14. 190103367
15. 190107357
16. 190116761
17. 190117791
18. 190118529
19. 190120791
20. 190123631
21. 190125036
22. 190127896
23. 190129996

In total, there are 6,003 cases in your dataset where the narrative includes a mention of the absence of a helmet.

You

Looking at the original csv file, for which case_numbers does Narrative_1 not mention helmet(s) or mention helmet use unknown? Text in Narrative_1 to help identify this could include language such as but not limited to: 'UNSURE IF HELMET', 'UNSURE IF HELMETED', 'UNSURE IF PT WEARING HELMET', 'UNSURE IF PT WEARING A HELMET', 'UNSURE IF PT WAS WEARING HELMET', 'UNSURE IF PT WAS WEARING A HELMET', 'UNKNOWN IF HELMET', 'HELMET UNKNOWN', 'UNKNOWN HELMET', 'NO MENTION OF HELMET', 'UNK HELMET', 'HELMET UNK', 'HELMET NS', 'NS HELMET', ‘?HELMET’, ‘? HELMET’, ‘HELMET?’
2 / 2

© 2024 Burford KG et al. *JAMA Network Open.*

ChatGPT
ChatGPT
I have identified two sets of case numbers based on the narrative's mention of helmets:

24. **Cases where helmet use is uncertain or unknown:**
    - First 10 case numbers: 190110570, 190119860, 190123339, 190129926, 190201469, 190207661, 190236938, 190249335, 190305066, 190328681.
    - Total number of such cases: 828.
25. **Cases where there is no mention of a helmet (excluding cases already identified as with or without helmet):**
    - First 10 case numbers: 190103292, 190103345, 190103420, 190103432, 190104427, 190105465, 190105512, 190105523, 190105528, 190106393.
    - Total number of such cases: 43,598.

These results help categorize the cases based on helmet usage or the lack of information regarding helmet usage in the narratives.

You
Could you make a csv file with cpsc_case_number, Narrative_1, and new column "helmet status". Based on their Narrative_1 descriptions and criteria above categorize each case number "helmet not mentioned" or "not wearing helmet" or "wearing helmet" for all case_numbers. There should be no duplicate case numbers. Should be one-to-one. Double check for mistakes
ChatGPT
Error analyzing
It appears that there are duplicate **cpsc_case_number** entries in the dataset. I will first remove these duplicates to ensure a one-to-one mapping between **cpsc_case_number** and **helmet_status**. Let's proceed with this correction and then create the CSV file as requested.
The CSV file has been created with each **cpsc_case_number**, its corresponding **narrative_1**, and the newly added **helmet_status** column. The data is categorized based on whether the narrative mentioned "helmet not mentioned," "not wearing helmet," or "wearing helmet." Duplicates have been removed to ensure a one-to-one correspondence.
You can download the file using the link below:
Download the CSV File

© 2024 Burford KG et al. *JAMA Network Open.*

December 12, 2023 (Intermediate Detail Prompt)

ChatGPT
You've uploaded a file named "df_helmet.csv". How can I assist you with this file?
You
for every cpsc_case_number there is a narrative_1 with patient description and details of a patient accident that involved an injury. based on their narrative_1 descriptions, can you use the following criteria to create a new variable called “helmet_status”, which is generated by categorizing each case number into one of the following categories "helmet not mentioned" or "not wearing helmet" or "wearing helmet" using these criteria:
"helmet not mentioned”: cpsc_case_numbers where narrative_1 mentions that helmet use was unknown or that the term “helmet” was not recorded within narrative_1. the narrative_1 column could include any variation of the following phrases: "unsure if helmeted", "unknown if helmeted", "no mention of helmet", or "?helmet". please consider that there could be other phrases that indicate unknown helmet use.
“not wearing helmet”: cpsc_case_numbers where narrative_1 mentions that the case number/patient was not wearing a helmet at any point. the narrative_1 column could include any variation of the following phrases: "no helmet" or "without helmet" or "not wearing helmet" or "unhelmeted". please consider that there could be other phrases that indicate the patient was not wearing a helmet.
“wearing helmet”: cpsc_case_numbers where narrative_1 mentions that the case number/patient was wearing/had a helmet at some point. the narrative_1 column could include any variation of the following phrases: "with a helmet" or "had a helmet" or "cracked helmet" or "+ helmet". please consider that there could be other phrases that indicate the patient was wearing a helmet. can you make a csv file with cpsc_case_number, narrative_1, and new column "helmet status" based on the criteria above?
ChatGPT
The dataset contains columns for **cpsc_case_number** and **narrative_1**. I will now create a new column, **helmet_status**, based on the criteria you provided. This column will categorize each case number into one of the following categories: "helmet not mentioned", "not wearing helmet", or "wearing helmet". Let's proceed with the categorization.
The updated CSV file, which includes the new column **helmet_status** based on the criteria you provided, has been created. You can download it using the following link.

© 2024 Burford KG et al. *JAMA Network Open.*
