# Supplementary material for: Use of Generative AI to Identify Helmet Status Among Patients With Micromobility-Related Injuries From Unstructured Clinical Notes
Source: JAMA Netw Open. 2024 Aug 13;7(8):e2425981. doi: 10.1001/jamanetworkopen.2024.25981 (PMC11322845; doi:10.1001/jamanetworkopen.2024.25981)
Supplement: Supplement 2. — Data Sharing Statement [file jamanetwopen-e2425981-s002.pdf]

Data Sharing Statement

Burford. Use of Generative AI to Identify Helmet Status Among Patients With Micromobility-Related Injuries From Unstructured Clinical Notes. *JAMA Netw Open*. Published August 13, 2024. doi:10.1001/jamanetworkopen.2024.25981

Data
Data available: yes
Data types: deidentified participant data, data dictionary
How to access data: data is deidentified, publicly available at the NEISS query builder here: https://www.cpsc.gov/cgibin/neissquery/home.aspx the data dictionary for NEISS is here:https://www.cpsc.gov/s3fs-public/January-2022-CPSC-Only-NT-NEISS-Coding-Manual.pdf?VersionId=KSUolSeeOpWoGAXtq0Zb_EVaHSiOdzFe the data and code will be provided upon publication on Zenodo at the following link: https://doi.org/10.5281/zenodo.12724290.
When available: With publication

Supporting Documents
Document types: statistical/analytic code
How to access documents: code will be made available on Zenodo upon publication
When available: with publication

Additional Information
Who can access the data: anyone requesting the data
Types of analyses: for any purposes
Mechanisms of data availability: with investigator support
